# Reduction of Surface Roughness by Means of Laser Processing over Additive Manufacturing Metal Parts

**DOI:** 10.3390/ma10010030

**Published:** 2016-12-31

**Authors:** Vittorio Alfieri, Paolo Argenio, Fabrizia Caiazzo, Vincenzo Sergi

**Affiliations:** Department of Industrial Engineering, University of Salerno, 84084 Fisciano, Italy; pargenio@unisa.it (P.A.); f.caiazzo@unisa.it (F.C.); sergi@unisa.it (V.S.)

**Keywords:** laser processing, surface roughness, additive manufacturing

## Abstract

Optimization of processing parameters and exposure strategies is usually performed in additive manufacturing to set up the process; nevertheless, standards for roughness may not be evenly matched on a single complex part, since surface features depend on the building direction of the part. This paper aims to evaluate post processing treating via laser surface modification by means of scanning optics and beam wobbling to process metal parts resulting from selective laser melting of stainless steel in order to improve surface topography. The results are discussed in terms of roughness, geometry of the fusion zone in the cross-section, microstructural modification, and microhardness so as to assess the effects of laser post processing. The benefits of beam wobbling over linear scanning processing are shown, as heat effects in the base metal are proven to be lower.

## 1. Introduction

**Figure 1:**
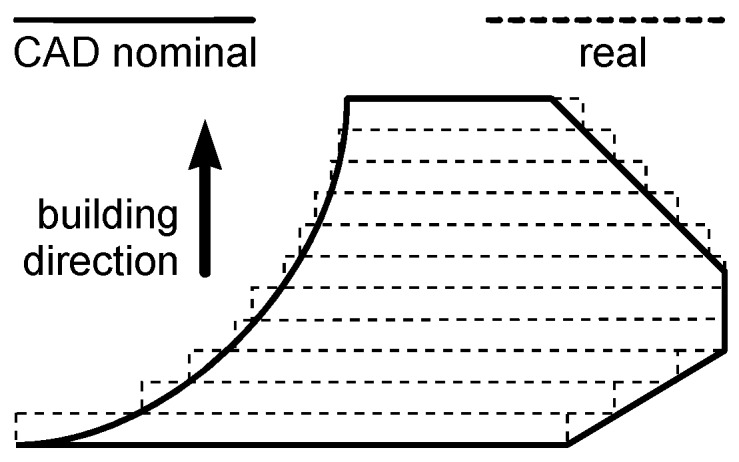
Staircase effect on the nominal surface in selective laser melting upon layering.

Additive manufacturing is receiving increasing interest in a wide range of industrial applications. In particular, new possibilities in lightweight design and direct fabrication of functional end-use parts are offered by selective laser sintering and melting of metal powders by means of laser irradiation [1,2]. Extensive research, experimental trials, and computational prediction are aimed at optimization of the processing parameters and the exposure strategies to set up the process [3,4]; nevertheless, surface quality may limit the application of the part if compared with conventional metal manufacturing processes such as machining. Namely (as for any additive layer manufacturing), since the Computer Aided Design (CAD) model of the object is preliminarily sliced into layers, the resulting contour of the real part is a stepped approximation of the nominal surface; it has been proved [5] that a staircase effect is induced (Figure 1) depending on both the local theoretical curvature and the sloping angle with the building direction. Although the thickness of the building layers can theoretically be reduced to improve surface finish, a threshold of minimum slicing is given by the average powder grain size. A distinct lay pattern (i.e., a distinct directional feature) is hence produced on the surface, depending on the building direction. Surface tension governing wetting is also a factor; hence, flat-built parts are also affected on the up-skin. Further unevenness results on overhanging surfaces, due to either dross formation or removal of the supporting structures. Because of these—depending on the technology and the average powder grain size—standards for surface finish may not be evenly matched on a single complex part. Depending on the manufacturer and the powder size, arithmetic as-built roughness usually ranges from 8 to 20 μm [6], whereas tighter standards could be required [7].

Therefore, post processing treating for the purpose of surface modification in terms of morphology and roughness is required. Several methods can be considered: computer numerical control (CNC) machining, shot peening, sandblasting, and infiltrating are suggested [8]; some are deemed to be unsuitable for local improvement on complex shapes, some are not fit for the purpose of generating different surface features on the same component, and some are not capable of reliable monitoring and automation.

When finishing is instead driven by a laser beam, laser surface modification (LSM) is in place: namely, surface peaks are melted to fill the valleys, resulting in a smoother surface, provided that overmelting is prevented [9]. Depending on the laser operation mode, two processes are reported [10]: macropolishing with continuous wave emission and micropolishing with pulsed radiation. As a consequence of tight focusing, laser energy is effectively delivered where required, thus suitably affecting the surface and preventing uncontrolled thermal penetration, distortion of the base metal, and thermal stresses leading to possible cracking and fatigue failure; furthermore, non-contact processing and automation are allowed. Nevertheless, the laser beam in heat treating is partially reflected, thus the absorbed energy and the eventual response depend on the surface type [11]. Hence, for the starting surface texture: the higher the starting roughness, the lower the reflectivity, and the intensity distribution and pulse duration are also involved [12] in metals.

It has been shown [6,9,13] that the main parameter in macropolishing is energy density *E_s_* depending on delivered power *P*, focus diameter *D*_0_, and processing speed *s*:

(1) $$E_{s}=\frac{P}{D_{0}\cdot s}$$

An energy density on the order of 30 J/mm^2^ has been proven to be effective [9] in reducing the roughness by at least 80% over metal sintered parts of bronze-infiltrated stainless steel; similar values have been considered in optimizing post-processing on 316 L stainless steel [6]. On the same subject, the possibility of laser polishing within the building machine and upon removal of surrounding non-melted powder has been investigated [14] using an energy density ranging from 1 to 10 J/mm^2^, resulting in a reduction of 70% at most on 316 L stainless steel. Alternation between manufacturing and polishing has been proposed [15] to address non-accessible surfaces in the building machine. Nevertheless, laser polishing in the same machine is deemed to be easily feasible as a final step of building when manufacturing is conducted by means of powder injection (i.e., laser metal deposition) instead of powder bed [16].

**Figure 2:**
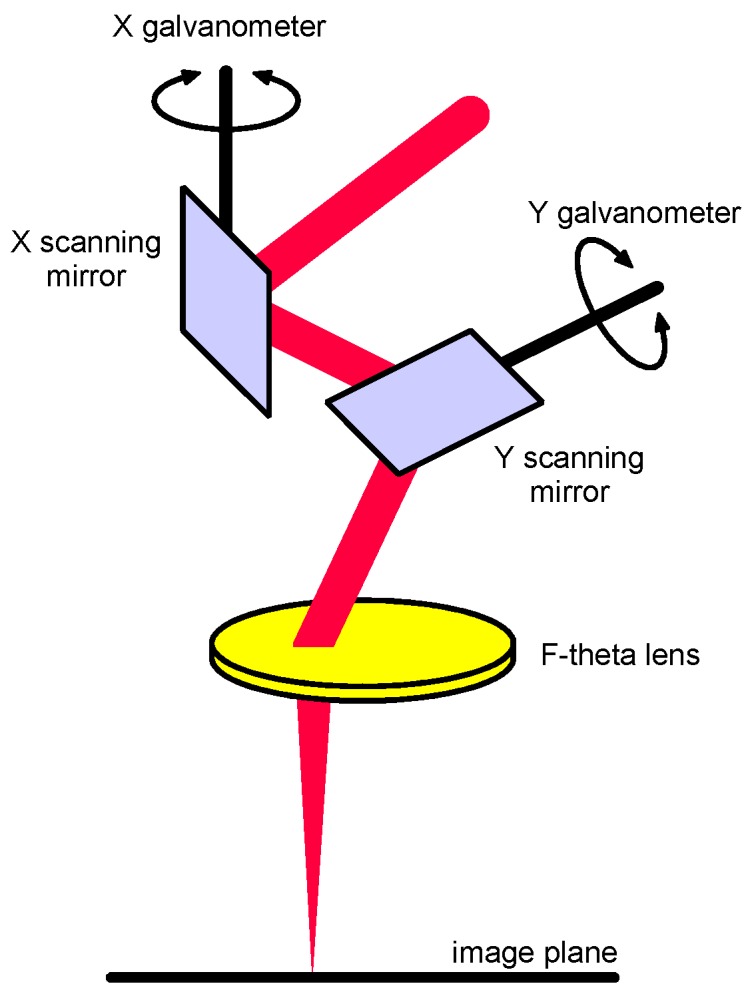
Base components and principle of laser scanning head.

It is worth noting that LSM by means of scanning optics can be performed effectively. Galvanometers moving laser-grade mirrors with low mass and inertia are arranged to deflect the laser beam in two dimensions [17] so as to conveniently position the focus on the work-piece (Figure 2), although joined mechanical and optical positioning and focus adjustment are required over 3D parts. To provide uniform irradiance and scanning rate across the focal plane, an F-theta lens must be considered; with respect to standard flat-field scanning lenses, the need for complex electronic correction of the scanning speed is prevented. Higher speed, optimized exposure strategies, and larger working distances are allowed, in addition to general advantages of laser material processing with robot-moved laser heads; accuracy and the capability to address complex 3D geometries are benefits. Given these reasons, processing via scanning optics is a subject of considerable interest both in research and industry to perform laser cutting, engraving, marking, and surface finishing [18].

**Figure 3:**
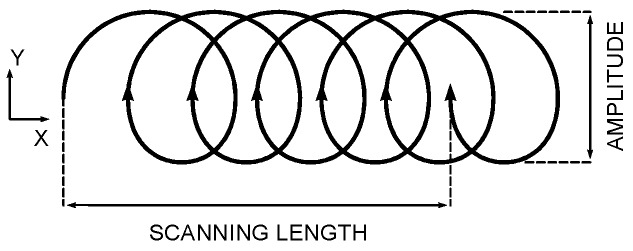
A prolate trochoid as a result of laser beam wobbling along the scanning path.

Wobbling of the laser beam along the scanning path is also allowed (Figure 3). A prolate trochoid is set—it being particularly helpful in welding—for the purpose of better covering the gap or eventual fixing of possible imperfections [19]. In the frame of LSM, beam wobbling is deemed to be a valid tool to widen the scanning traces, thus reducing the overall processing time on the surface to be polished. Defocusing would also result in increased width of the scanning traces [11]; nevertheless, this would require increased beam power or reduced processing speed for a given optimum energy density.

For a given processing speed *s*, the trace width is given by the wobble amplitude *A*, while the longitudinal step between two consecutive loops is only driven by wobble frequency *f* according to equations:

(2) $$X=s\cdot t+\frac{A}{2}\cos(2\pi ft)$$

(3) $$Y=\frac{A}{2}\sin\left(2\pi ft\right)$$

Based on these, it is worth noting that *s* results as mean processing speed along the scanning length.

LSM to improve surface topography by means of laser beam wobbling and linear scanning is discussed in this paper; a comparison is given in terms of roughness, geometry of the fusion zone, microstructural modification, and Vickers microhardness in the cross-section in order to assess the effectiveness of the process. Namely, as the starting roughness resulting from additive manufacturing depends on the sloping angle with the building direction, flat-, 45°-, and upright-built samples have been considered in order to test post-processing against different surface conditions. The operating window for the experimental plan has been found based on preliminary trials, a factorial design has been arranged, the main governing factors of beam laser wobbling being the wobble amplitude *A* to be set to 1 and 2 mm, the wobble frequency *f* to be set to 200 and 400 Hz, the building orientation of the sample as categorical factor. Linear scanning has also been performed to compare the results. Irrespective of the scanning strategy, LSM has been conducted at 1 kW operating laser power in continuous wave emission mode at 2 m∙min^−1^ scanning speed, which is intended to be the processing linear speed in the case of linear macropolishing, and the mean speed in case of a circular wobble path. A focused beam, 1 mm in diameter, has been delivered to the surface.

## 2. Results

### 2.1. Starting Roughness for As-Built Samples

**Figure 4:**
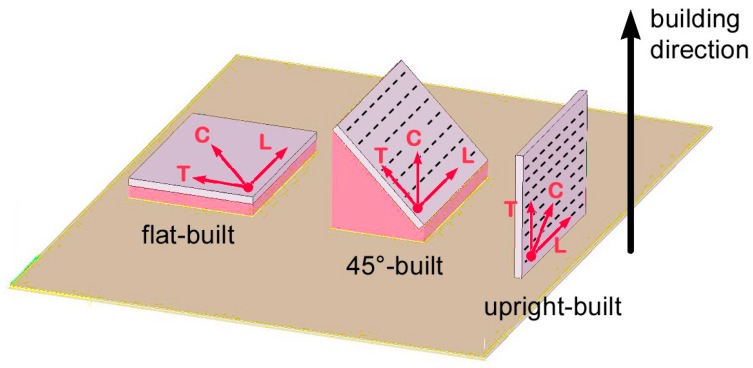
Manufacturing of the samples via selective laser melting; flat, 45°, and upright building.

Arithmetic roughness *R_a_* and peak-to-valley height *R_z_* have been measured on as-built samples either in longitudinal (L), transverse (T), and crossed (C) directions with respect to layering (Figure 4), so as to investigate any possible directional feature on as-built samples before LSM processing. It is worth noting that since no lay patterns are expected on flat build samples—the surface being formed by a single building layer—longitudinal and transverse scanning directions are intended to be mere directions of the sample sides in this case.

**Figure 5:**
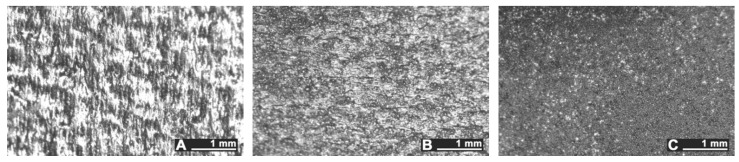
Surface texture, as-built samples: (**A**) flat-built; (**B**) 45°-built; and (**C**) upright-built.

Surface texture and resulting roughness clearly depend on the sloping angle with the building direction (Figure 5); namely, average roughness is higher for 45°- and upright-built samples (Table 1). Nevertheless, thin layering led to uniform surfaces, preventing any clear main pattern in terms of mean spacing of profile irregularities; hence, no deviation is found among longitudinal, transverse, and crossed roughness. As a consequence, a single average reference value for starting roughness is considered in the following for each given building direction to assess the effectiveness of LSM.

### 2.2. Roughness and Geometry of the Fusion Zone upon LSM

**Figure 6:**
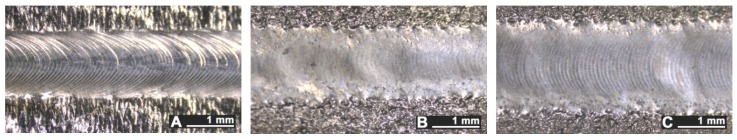
Examples of visual inspections upon laser surface modification (LSM) with laser beam wobbling, 2 mm amplitude at 400 Hz frequency: (**A**) flat-built; (**B**) 45°-built; and (**C**) upright-built samples.

**Figure 7:**
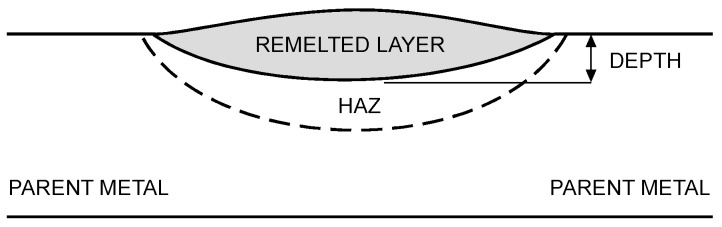
Scheme for width and depth of the remelted layer in the cross-section. HAZ: heat-affected zone.

Based on visual inspections upon LSM (Figure 6), shielding is deemed to be effective. Furthermore, no cracks or macropores resulted from any of the processing conditions (Table 2). Surface modification of each texture are then discussed in terms of percentage reduction of roughness Δ*R*_%_; the depth of the fusion zone (i.e., the remelted layer, Figure 7) with respect to the nominal surface in the cross-section has also been considered as response (Table 3); due to shading boundaries, the depth of the heat-affected zone (HAZ) should instead be discussed via microhardness testing.

Decreased roughness resulted in all conditions of the experimental plan; therefore, overmelting of the surface—which would increase roughness due to improper energy density—was prevented. Namely, major improvements were achieved when considering 45°- and upright-built samples, the process being capable of reducing *R_a_* below 2 μm under certain processing conditions. Two reasons are inferred. First, irrespective of the scanning strategy in LSM, a dependence of absorption on the surface type is assumed: the higher the starting roughness, the lower the reflectivity, the more effective the overall process; moreover, as LSM is driven by the melting of surface peaks, the higher they are, the better the response.

**Figure 8:**
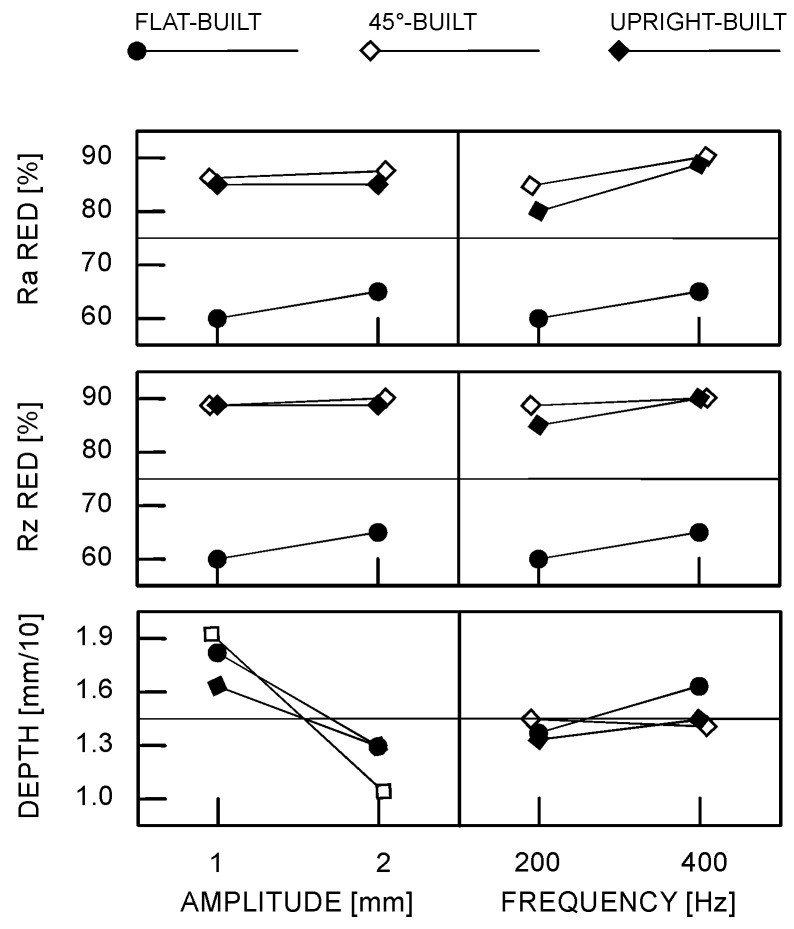
Main effects plots for roughness reduction percentage and depth of the remelted layer.

Further findings result from the discussion of the main effects plots (Figure 8) when referring to scanning in the case of laser beam wobbling. For a given building direction, both wobble amplitude and frequency have mild effects on either *R_a_* and *R_z_* roughness reduction. Interestingly, as a general rule, increasing wobble frequency for a given wobble amplitude results in decreasing roughness, but concurrent increasing depth of the remelted layer, as more loops per length are engraved along a given scanning length. Hence, the effect of heat accumulation in the metal is heavier; increasing wobble amplitude for a given wobble frequency also results in decreasing roughness, with concurrent decreasing depth of the fusion zone instead, since wider scanning traces are processed and overlapping among consecutive loops is affected with milder heat accumulation effects.

Since overheating and deterioration of bulk properties in the parent metal must be proven to be reasonable upon LSM, the suggestion of an optimum condition for processing would be pointless when based on a mere discussion of roughness reduction. Therefore, higher weight must be awarded to the technical constraint involving depth; as a consequence of this, although better polishing results from linear scanning in terms of roughness, wobbling with 2 mm amplitude at 200 Hz frequency is suggested within the current investigation domain.

### 2.3. Microstructure and Microhardness

**Figure 9:**
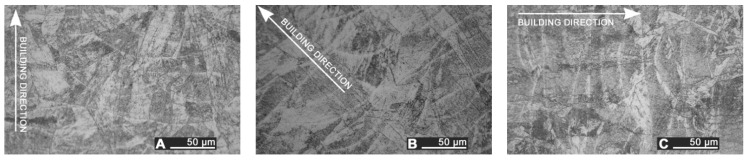
Melting pools in the cross-section: (**A**) flat-built; (**B**) 45°-built; and (**C**) upright-built samples.

**Figure 10:**
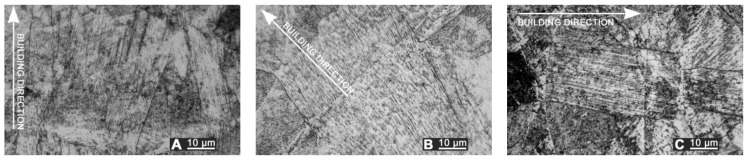
Micrographs, grain growth: (**A**) flat-built; (**B**) 45°-built; and (**C**) upright-built samples.

The depth of thermal penetration is worth investigating. In the unaffected parent metal, the appearance of lenticular-shaped melting pools is clear as a result of building and layer development (Figure 9); moreover, as heat flows toward the building plate during manufacturing, columnar growth along the building direction is shown in the magnified view (Figure 10).

In agreement with the literature [20], specific grain size and microstructure strongly depend on both the building strategy and the supporting structures; nevertheless, irrespective of these, a fully martensitic transformation is prevented due to nickel and chromium addition in the base powder, leading to large solidification undercooling and residual metastable austenite; moreover, tempering is promoted in the lowest layers during additive fabrication. Indeed, as-built samples are approximately composed of 70% mass fraction austenite and 30% martensite on average [21]; a reference microhardness of 265 HV is found, in agreement with similar results on the same alloy [22] and the material data sheet of the supplier.

**Figure 11:**
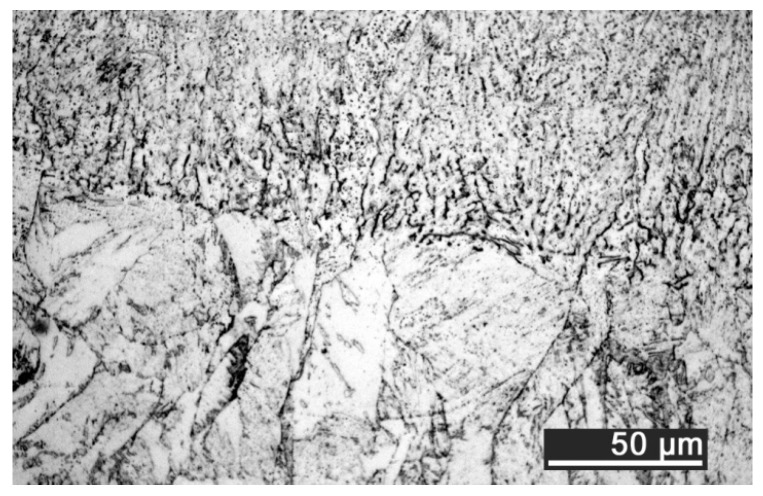
Heat-affected zone at the interface with the remelted layer; LSM with laser beam wobbling, 2 mm amplitude at 200 Hz frequency on flat-built sample.

As a consequence of LSM, softening to 235 HV on average is experienced in the fusion zone, austenite being retained as the main phase. Solution annealing is thought to be in place in the HAZ instead (Figure 11), based on referred metallographic analyses [23].

**Figure 12:**
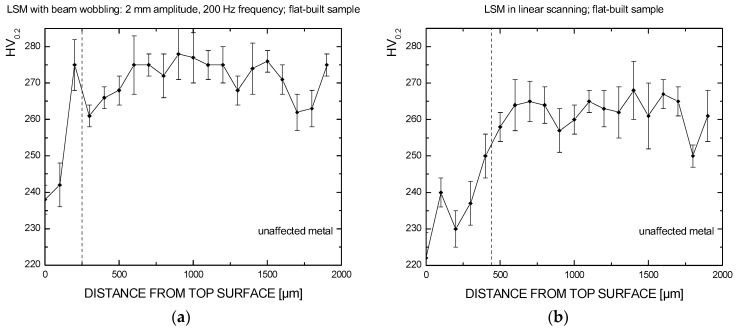
Vickers microhardness trend as a function of distance from top surface, as a result of LSM with beam wobbling (**a**) and LSM in linear scanning (**b**) on flat-built samples.

Namely, referring to the suggested condition for LSM with beam wobbling on flat-built samples, one may assume the parent metal is unaffected at an average depth of 250 μm, based on the trend of Vickers microhardness as a function of the distance from the nominal top surface; a depth of at least 400 μm is found instead when considering linear scanning with no wobble (Figure 12), although a dependence from the position on the building plate is inferred as reason for different hardness of the parent metal. The error bars are also given, based on three tests for each sample. Similar trends of microhardness have been found for 45°- and upright-built samples.

## 3. Discussion

Surface modification by means of laser beam is effective as a possible post processing treatment over stainless steel components resulting from additive manufacturing, in order to improve the surface topography. The response has been proven to depend on the starting features of the samples; namely, major improvements are achieved over 45°- and upright-built samples, the process being capable of reducing arithmetic roughness below 2 μm on average, thus matching the requirement for real parts in valid operating conditions. A less significant reduction to 5 µm has been shown in the literature [14], hence 70% with respect to initial roughness can be achieved within each single trace on stainless steel additive manufactured surfaces. Moreover, the results refer to polishing upon removal of non-melted powder within the building chamber, with consequent issues to accessing a general 3D part. Additionally, reduced spot sizes are allowed when polishing in the building machine, thus multiple passes would be required over a large surface.

A valuable reduction of roughness to 1.4 µm (on the order of 80%) has been achieved over stainless steel parts by using a custom-made hybrid re-cladding machine for a given processing speed ranging from 0.4 to 0.6 m∙min^−1^ [6]. Here, it has been shown that an increased speed of 2 m∙min^−1^—resulting in reduced processing time for a given surface extension—is possible. Even higher speed on the order of 3 m∙min^−1^ are reported in the literature [15], although up to 5 overlapped passes are required to properly smooth the surface below 1 µm.

Nevertheless, since a change in the microstructure is induced as a consequence of laser processing, the effects on the parent metal must be addressed. Specific benefits are offered when performing laser beam wobbling compared with linear scanning: heat effects are proven to be lower, based on the depth of the fusion zone as well as on the extent of the HAZ resulting from Vickers microhardness testing. For given operating power of 1 kW and processing speed of 2 m∙min^−1^, wobbling with 2 mm amplitude at 200 Hz frequency is suggested. With respect to the laser path, an oscillating beam had been proposed previously in the literature [24], resulting in a reduction of roughness up to 92% over AlSi10Mg additive manufactured parts, but a one-dimensional scanning system was used.

Interestingly, additional opportunities are offered, as the resulting features of the surfaces can be conveniently graded by means of proper setting of frequency and amplitude, with reliable monitoring from the laser source. For these reasons, grounds for application on real parts are given, although additional studies on overlapping traces must be conducted to perform the process over larger surfaces.

## 4. Materials and Methods

An EOSINT M270 commercial laser sintering system (EOS, Krailling, Germany) has been used to manufacture a suitable number of testing samples, 3 mm thick. Pre-alloyed, argon gas atomized virgin commercial EOS GP1 stainless steel powder, 20 μm mean grain size, corresponding to standard UNS S17400 chromium-copper precipitation hardening steel in terms of nominal chemical composition (Table 4) has been considered.

Processing power, speed, layer thickness, and hatching strategies are based on preliminary trials aimed to optimize selective laser sintering and full dense structure (Table 5); a nitrogen inert atmosphere has been arranged. Flat, 45°, and upright building orientations have been addressed with respect to the building plate in order to test the effectiveness of the post-processing scanning strategy against different surface conditions. Supporting structures were required on downward facing surfaces of overhanging samples as well as below flat-built samples, as a threshold angle was exceeded; nevertheless, roughness on the unsupported side was investigated. No post-processing or heat treating for stress relief were conducted upon fabrication before performing LSM.

To perform LSM, a prototype scanning optic was used with a fibre laser (Table 6), fibre delivered to a gantry processing station. Based on the equations of basic laser optics [11], a resulting scanning focus diameter *D*_0_ of approximately 1 mm is given by:

(4) $$D_{0}=\frac{\lambda \cdot F\cdot k_{G}\cdot M^{2}}{D_{in}}$$

λ being the operating nominal wavelength of the laser beam, *F* the effective focal length of the F-theta lens, *k_G_* the factor 4/π accounting for laser beam diffraction of the theoretical corresponding Gaussian beam, *M*^2^ the beam propagation parameter, and *D_in_* the diameter of the laser beam when entering the optics. For a given focus diameter of 1 mm, power and speed were conveniently set in the experimental plan, aiming to provide an energy density on the order of 30 J/mm^2^, which has been proven to be effective for the purpose of surface polishing, as discussed in the literature [6,13]. LSM in form of a single 50 mm long scanning trace—the laser beam being normal with respect to the surface of the sample—has been performed; replications of each LSM testing condition have been considered to average the responses.

As a carryover of a prior patented device [25], a diffuser has been developed for inert shielding to the mere purpose of this research; argon has been supplied at a constant flow rate (40 L/min), at atmospheric pressure.

As a consequence of slicing along the building direction, it is worth noting that a lay pattern would be expected over 45°- and upright-built samples; therefore, measuring traces to assess the starting roughness before LSM should be taken at a right angle to the main lay; hence, the same angle should be considered for LSM processing, and the same should be taken for measurements upon LSM as well. A contact stylus arm operating in a transverse range displacement of 1 mm with a conical 30° needle tip has been used, the stylus being moved at a speed of 1 mm∙s^−1^ over the surfaces by a transverse unit. All of the measurements were conducted in compliance with the ISO standard for surface roughness testing [26]; the results are averaged among at least three traces to assess statistical significance.

Measurements on the responses in terms of heat effects have been conducted by means of conventional optical microscopy and Vickers microhardness testing; an indenting load of 0.200 kg has been used for a dwell period of 10 s; a step of 100 μm has been allowed between consecutive indentations, in compliance with ISO standard [27] for hardness testing on metallic materials.

## 5. Conclusions

Based on the results of the experimental plan, laser surface modification has been proven to be feasible to the purpose of reducing the surface roughness resulting from additive manufacturing. As a consequence, one may assume these findings would give grounds for further exploitation of additive manufacturing in a number of technical applications where common standards may not be properly matched, currently.

Namely, additional advantages in comparison with conventional machining are offered by the possibility of wobbling of the laser beam instead of linear scanning, as limited thermal affection is benefited.

## Figures and Tables

**Table 1:** Longitudinal, transverse, and crossed average as-built roughness.

Building Mode	Measuring Direction	Arithmetic Roughness *R_a_*		Peak-to-Valley Height *R_z_*	
		Average (μm)	Std. Deviation (μm)	Average (μm)	Std. Deviation (μm)
Flat-built	Longitudinal	6.06	0.60	34.33	3.59
	Transverse	6.87	0.74	35.50	3.10
	Crossed	7.44	0.10	43.50	3.15
45°-built	Longitudinal	14.40	0.70	106.80	6.77
	Transverse	15.07	0.55	111.30	3.54
	Crossed	14.83	1.17	109.27	12.65
Upright-built	Longitudinal	16.20	1.75	107.47	12.82
	Transverse	15.83	0.32	104.83	4.39
	Crossed	16.50	1.04	109.77	3.97

**Table 2:** Transverse cross-sections; 1 kW power, 2 m·min^−1^ scanning speed, 1 mm diameter focus.

Condition	Flat-Built Sample	45°-Built Sample	Upright-Built Sample
Beam Wobbling: 1 mm Amplitude, 200 Hz Frequency	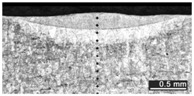	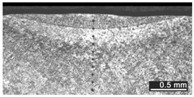	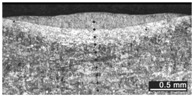
Beam Wobbling: 1 mm Amplitude, 400 Hz Frequency	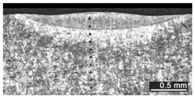	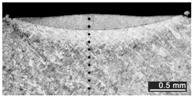	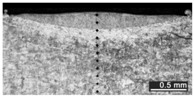
Beam Wobbling: 2 mm Amplitude, 200 Hz Frequency	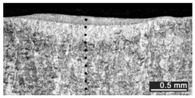	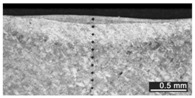	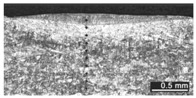
Beam Wobbling: 2 mm Amplitude, 400 Hz Frequency	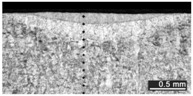	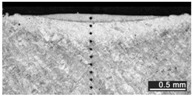	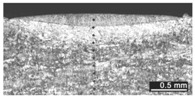
Linear Scanning	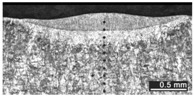	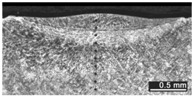	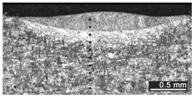

**Table 3:** Responses for each processing condition.

Building Mode	Conditions		Arithmetic Roughness *R_a_*			Peak-to-Valley Height *R_z_*			Depth (mm)
	*A* (mm)	*f* (Hz)	Average (μm)	Std. Dev. (μm)	Δ*R*_%_	Average (μm)	Std. Dev. (μm)	Δ*R*_%_	
Flat-built	1	200	3.09	0.19	54	17.11	2.06	55	0.17
	1	400	2.26	0.19	67	13.66	0.78	64	0.19
	2	200	2.27	0.21	67	12.81	0.21	66	0.12
	2	400	2.54	0.16	63	13.15	0.61	65	0.14
	Linear scanning		1.73	1.21	74	9.35	4.62	75	0.18
45°-built	1	200	2.66	0.31	84	13.85	1.49	87	0.19
	1	400	1.92	0.32	88	10.59	1.01	90	0.19
	2	200	1.91	0.24	88	11.12	0.94	90	0.12
	2	400	1.85	0.37	89	10.38	1.68	90	0.10
	Linear scanning		1.34	0.26	92	7.41	1.30	93	0.19
Upright-built	1	200	3.11	0.12	79	16.67	0.61	85	0.17
	1	400	1.69	0.33	89	10.38	2.30	90	0.16
	2	200	2.59	0.15	82	14.55	1.15	87	0.11
	2	400	1.71	0.32	88	9.47	1.26	91	0.15
	Linear scanning		1.54	0.27	90	9.16	0.79	92	0.19

**Table 4:** Nominal composition (wt. %) of the powder; single values to be intended as maximum.

Cr	Ni	Cu	Mn	Si	Mo	Nb	C	Fe
15.0 ÷ 17.5	3 ÷ 5	3 ÷ 5	1	1	0.5	0.15 ÷ 0.45	0.07	balanced

**Table 5:** Main features and processing parameters in selective laser melting of stainless steel powder.

Gain Medium	Fibre, Ytterbium Doped YAG
Operating laser power (W)	195
Linear processing speed (m∙s^−1^)	1
Hatch spacing (mm)	0.10
Layer thickness (μm)	20
Focused spot diameter (μm)	90

**Table 6:** Laser source and scanning optics for LSM, main technical data.

Gain Medium	Fibre, Yb:YAG
Operating nominal wavelength (nm)	1030
Beam parameter product (mm × mrad)	6.0
Beam propagation parameter (/)	17.8
Core diameter of the delivering fibre (mm)	0.300
Diameter of the laser beam entering the optics (mm)	25
Scan head aperture (mm)	35
Effective focal length (mm)	1000
Image field (mm × mm)	400 × 400
